# Synergism and Rules from Combination of *Baicalin, Jasminoidin* and *Desoxycholic acid* in Refined Qing Kai Ling for Treat Ischemic Stroke Mice Model

**DOI:** 10.1371/journal.pone.0045811

**Published:** 2012-09-26

**Authors:** Jian Li, Run-guo Wu, Fan-yun Meng, Zhong Wang, Chang-ming Wang, Yong-yan Wang, Zhan-jun Zhang

**Affiliations:** 1 School of Basic Medical Science, Beijing University of Chinese Medicine, Beijing, People's Republic of China; 2 State Key Laboratory of Cognitive Neuroscience and Learning, Beijing Normal University, Beijing, China; 3 School of Resources Science & Technology, Beijing Normal University, Beijing, China; 4 Institute of Basic Research in Clinical Medicine, China Academy of Traditional Chinese Medicine, Beijing, China; Massachusetts General Hospital/Harvard Medical School, United States of America

## Abstract

Refined Qing-Kai-Ling (QKL), a modified Chinese medicine, consists of three main ingredients (Baicalin, Jasminoidin and Desoxycholic acid), plays a synergistic effect on the treatment of the acute stage of ischemic stroke. However, the rules of the combination and synergism are still unknown. Based on the ischemic stroke mice model, all different kinds of combination of Baicalin, Jasminoidin, and Desoxycholic acid were investigated by the methods of neurological examination, microarray, and genomics analysis. As a result, it confirmed that the combination of three drugs offered a better therapeutical effect on ischemic stroke than monotherapy of each drug. Additionally, we used Ingenuity pathway Analysis (IPA) and principal component analysis (PCA) to extract the dominant information of expression changes in 373 ischemia-related genes. The results suggested that 5 principal components (PC1-5) could account for more than 95% energy in the gene data. Moreover, 3 clusters (PC1, PC2+PC5, and PC3+PC4) were addressed with cluster analysis. Furthermore, we matched PCs on the drug-target networks, the findings demonstrated that Baicalin related with PC1 that played the leading role in the combination; Jasminoidin related with PC2+PC5 that played a compensatory role; while Desoxycholic acid had the least performance alone which could relate with PC3+PC4 that played a compatible role. These manifestations were accorded with the principle of herbal formulae of Traditional Chinese Medicine (TCM), emperor-minister-adjuvant-courier. In conclusion, we firstly provided scientific evidence to the classic theory of TCM formulae, an initiating holistic viewpoint of combination therapy of TCM. This study also illustrated that PCA might be an applicable method to analyze the complicated data of drug combination.

## Introduction

Stroke, one of the most common neurological disorders and the third leading cause of death in the worldwide (ranking behind heart disease and all forms of cancer), can be classified into two major categories: ischemic and hemorrhagic [1]. Due to 87% of strokes are caused by ischemia, it has become the focus of many researchers. However, there is no routine effective, generally accepted and specific treatment for ischemic stroke yet, except for aspirin and thrombolytic treatment with recombinant tissue plasminogen activator for highly selected patients [2], [3].

Nevertheless, stroke is much more complex than initially anticipated because it is often caused by multiple molecular abnormalities, rather than being the result of a single effect. For this reason, it is huge challenge for specific-target drug or mono-therapy to impact on many aspects of clinical trials. In this regard, the application of combinational drugs or polypill, in which two or more drugs interact with multiple targets simultaneously, is considered as a rational and efficient form of therapy designed to control complex diseases such as stroke[4]–[6].

**Figure 1:**
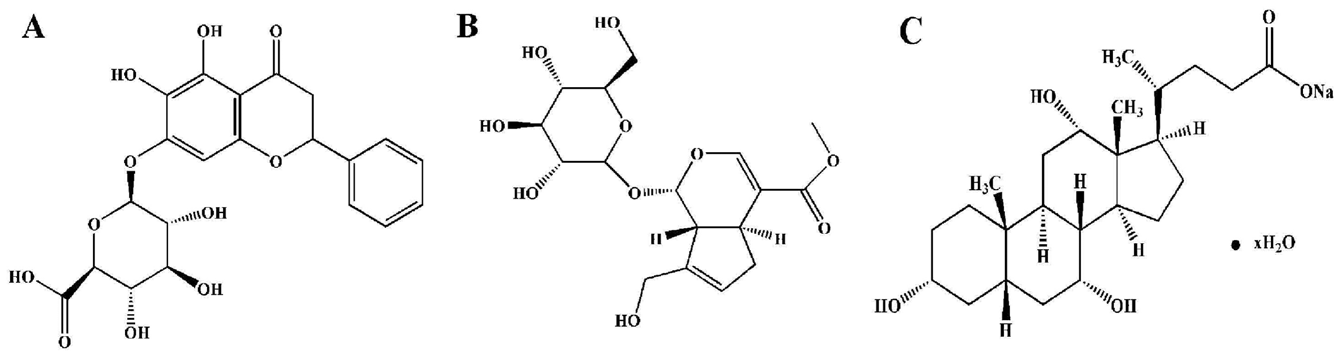
The structures of baicalin, jasminoidin and desoxycholic acid. (a) Baicalin. (b) Jasminoidin. (c) Desoxycholic acid.

Interestingly, Traditional Chinese Medicine (TCM), from the viewpoint of holism, always advocates drug-combined administrations. Over thousands of years, prescriptions of TCM called as formulae are made by practitioners according to their experience and heritage from ancestors [7]. We and other researchers demonstrated that some TCM preparations (e.g. Qing-Kai-Ling, Xue-Se-Tong, and Dan-Shen, etc.) showed effectiveness for the treatment of stroke by anti-oxidation, anti-inflammation, protecting against ischemic reperfusion injury, and enhancing the tolerance of ischemic tissue to hypoxia [8]–[10]. Among them, Qing-Kai-Ling (QKL) was from a modifying traditional Chinese medicine, An-Gong-Niu-Huang Pill. It was widely used in clinical to treat acute stages of cerebrovascular diseases [11]–[13], inflammatory related diseases, and so on [14]–[16]. Considering the complex components, quality stabilization, and clinical safety, the refined QKL injection was developed to aim at acute ischemic stroke for 20 years [13], [17]. Refined QKL injection consists of Baicalin, Jasminoidin and Desoxycholic acid (Fig. 1), which proved effective for the treatment of ischemic stroke through protecting neurological impairment and secondary lesions [18]. However, the studies of combinational rules and roles of each ingredient in QKL have not been reported.

In present study, we used methods of neurological examination, histochemistry and microarray to compare the efficacy of various kinds of combined and single forms of 3 ingredients to treat cerebral ischemia mouse model. Moreover, we attempted several mathematics algorithms and systems biology analysis methods to reveal the rules of combination of drugs.

## Results

### The effect of drug individual and combinations on neurological deficit and ischemic infarct

**Figure 2:**
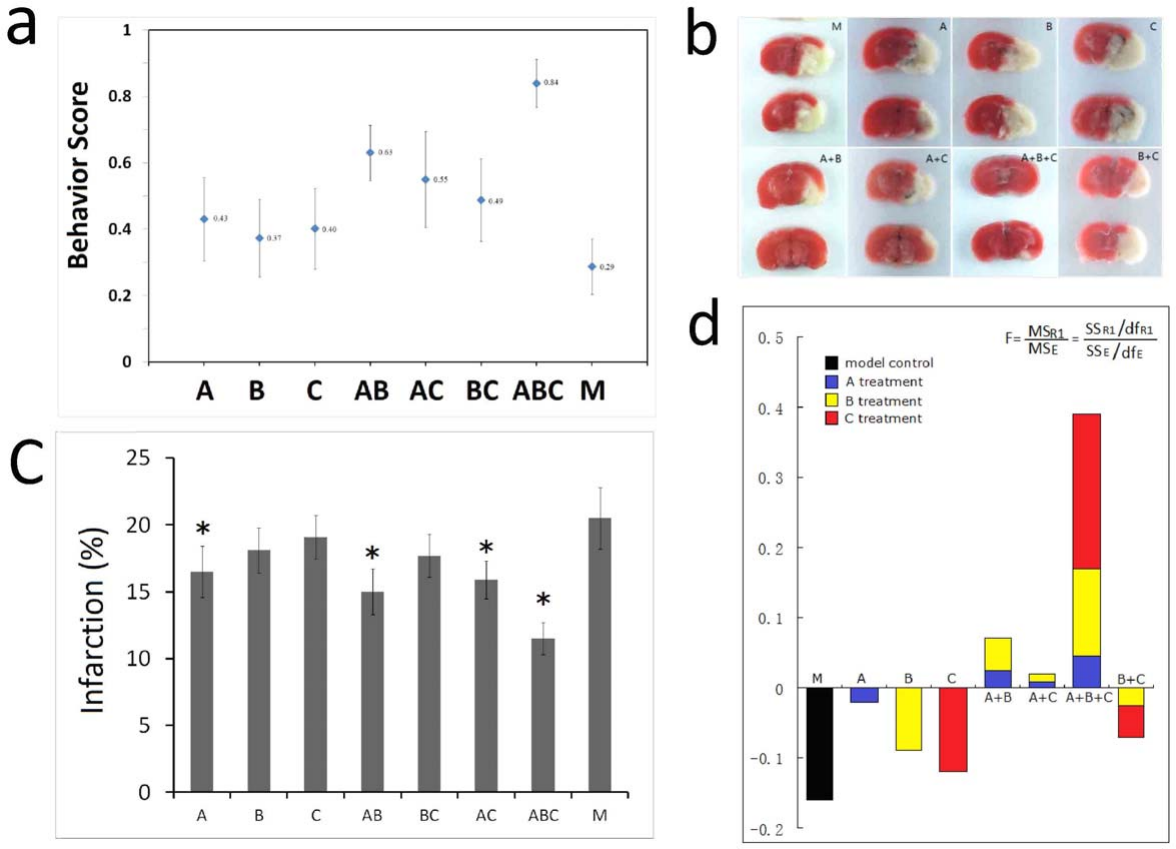
The results of pharmacodynamic effect on MACO. (a) The neurological examination scores (behavior test). According to the principle of the neurological examination, a lower score means a worse pathological condition. The score of each experimental group came from the average of sixteen subjects' behavioral performances. (b) The TTC staining results. A higher proportion of cerebral infarct means a worse ischemic injury. The same sixteen subjects as those in neurological examination for each experimental group were used. (c) The image analysis results. A higher proportion of cerebral infarct means more severe in ischemic injury, n = 16, *mean ±SE*. *p<0.05, **p<0.01 vs model control. (d) The results of factorial analysis on drug A, B and C.

We firstly investigated the behavior scores at 3 hrs and 24 hrs after MCAO to evaluate the beneficial effects of the treatment with the various combined drugs. The results showed the behavior scores was not significant difference between each group at 3 hrs after reperfusion (data not show), while at 24 hrs, the scores of ABC and AB treatment group are significantly higher than model group (M) (Fig. 2a). The cerebral infarct volumes were later on measured by computer image analysis of TTC-stained 2 mm thick brain sections. The results showed that in drug A, AB, AC, and ABC treated mice, the percentage of infarct volume was significantly reduced than that in model control mice (Fig. 2b). Image analysis and statistical results showed that the drug treated groups except C had significant lower infarct volumes than M and ABC was lower than any other groups (Fig. 2c). Factorial analysis demonstrated that the factor A was the primary contribution for the efficacy of the drugs (Fig. 2d).

### Drug individual and combinations match different bio-network and biology functions

The present mRNA microarray had 373 ischemic stroke related genes. Three mice of each group were sampled and each sampling was repeated four times in the microarrays. Consequently, twelve parallel outcomes of one gene in one group were observed and means of all the parallel outcomes were used to describe the genes' performances in different conditions. To validate the microarray results, real-time polymerase chain reaction (qRT-PCR) was used to confirm the expression of selected genes, including F5, Sh2bpsm1, Il1a, Dusp4, Rgs6 and Gpx2. The outcome of qRT-PCR indicated that broad, array-based gene expression measurements were reliable for determining gene expression patterns in the brain (figure S1).

**Figure 3:**
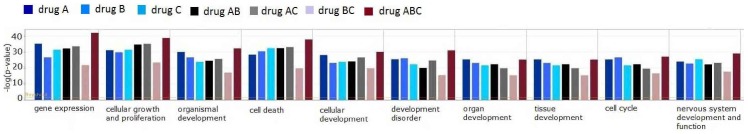
The Top 10 bio-functions analyzed by ingenuity pathway analysis ingenuity system (IPA).

Based on Ingenuity Pathways Knowledge base (IPA), 46 selected drug-target genes, fold = −1.5 to +1.5, were overlaid onto a global molecular network developed from information contained in the IPA base (table S1). Networks of these focus molecules were then algorithmically generated based on their connectivity. The results showed that about 32 bio-functions were affected by various drug combinations, the top 10 is in turn: 1) gene expression, 2) cellular growth and proliferation, 3) organismal development, 4) cell death, 5) cellular development, 6) development disorder, 7) organ development, 8) tissue development, 9) cell cycle, and 10) nervous system development and function. The functional analysis identified the probability (p-value), each biological function assigned to various combined drugs demonstrated that the AB, AC, and ABC combinations can addressed higher p-value than BC combination, which suggested the outcomes from gene expression profiles have correlations between scores from neuro-pathological examination (Fig. 3).

### Principal component analysis (PCA) to expose genes profiles and combinative effects of drugs

**Figure 4:**
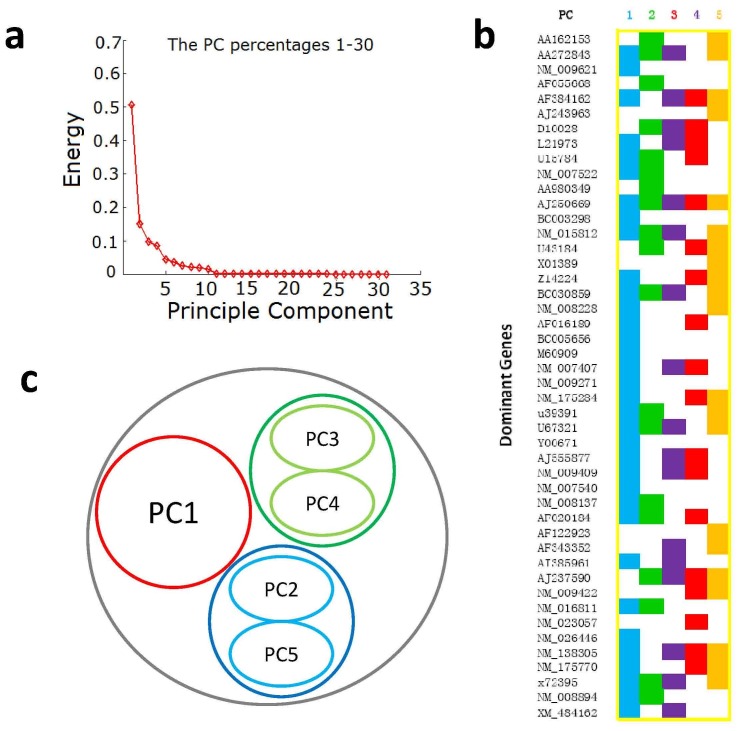
Principal component analysis of the gene expression profiles. (a) The Principal component analysis (PCA) energy ranking, in which the top five Principal components (PCs) account for >95% of total energy. (b) The distribution of the 373 genes in the five PCs, according to their normalized PCA values. Most of genes in each PC are located between ±0.2, while the minorities of genes outside this range are selected as dominant genes of a PC. (c) The clustering of PCs. Distributions of dominant genes shows that PC 2 and PC 5, and PC 3 and PC 4 have the similar mode, but PC 1 is unique.

**Figure 5:**
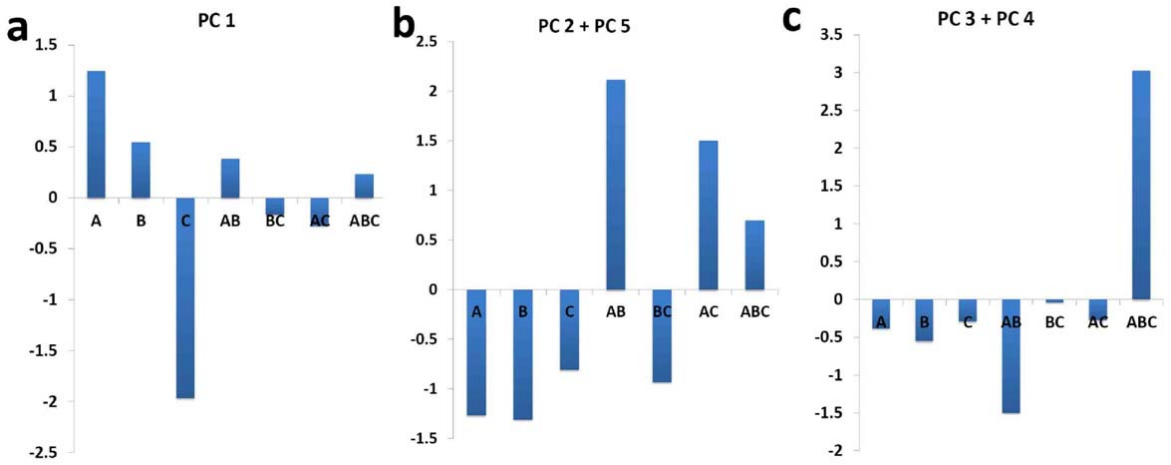
The association between values of each PCs with drugs combination. ***5a*** showed that drug A (Baicalin) matched PC 1, which offered a potency of the principal therapeutic effect. ***5b*** suggested that drug B (Jasminoidin) matched PC 2 + PC 5, which showed the supplementary role on pharmacodynamics. ***5c*** showed that drug C (Desoxycholic acid) only played some compatible effect on the contribution of pharmacodynamics.

To investigate the combinational effects of A, B and C, principal component analysis (PCA) was used to analysis gene expression profiles. Figure 4a showed that five components could account for more than 95% energy in the gene data without losing useful information. Every gene had a transformed score for a specified experimental group in a principal component (PC). From the distributions, ±0.2 could be appointed a cutoff to select the genes with the greatest contribution. Consequently, there were 50, 31, 24, 26 and 30 genes with a value above the threshold, respectively from PC1 to PC5 (Figure 4b, table S2). In total, 72 genes and 79 cell processes in any PC above this threshold were selected as targets. To analyze this result, we distinguished 52 (65.8%) processes playing a role in ischemic pathology and recovery from the total 79 processes, according to the known ischemic stroke process [19]–[21] and previous reports in ISI Web of Knowledge. In detail, PC1 accounted for most processes, a considerable part of which were exclusive, PC2 + PC5 had many shared processes and a few exclusive one, and PC3 + PC4 were evidently concentrated in high commonness classes (Figure 4c). In order to further unveil the relationship between each ingredient with PCs, we matched each PC on drug-target networks. The results showed that the drug A had the best performance in PC1 (Fig. 5a), drug B had similar performances with A related with PC2+PC5 (Fig. 5b), drug C evidently had the least performance alone, but it can be matched with PC3+PC4 (Fig. 5c).

## Discussion

Multi-component therapeutics, in which two or more agents interact with multiple targets simultaneously, is considered as a rational and efficient form of therapy designed to control complex diseases [4], [22]. Researchers hypothesize that synergism from combinations may be greater than the sum of the individual effects [6], [23]. Recently, some researchers have made good attempts in this explanation using approaches of system biology and network pharmacology to identify the combinational roles of multi-component therapeutics from Chinese medicines [24]. However, these researches still need to be verified through many experiments. We and others have shown that refined QKL offered protective effects on focal cerebral ischemia-reperfusion injury [9], [10], [13], [18], [25]–[27]. To further understand the synergistic effect and combinational rules, in present study, we designed seven combinations from three drugs to treat cerebral ischemia on mice MCAO models. From the TTC staining data and behavior scores, we found the AB, AC, and ABC combinations remarkably ameliorate brain damage. These initial results suggest the combinational drugs do offer better therapeutic effect on cerebral ischemia. In these experiments, Drug A, Baicalin, is a major flavonoid derived from and regarded as the active ingredient of a Chinese herbal, *Scutellaria baicalensis Georgi*. It proved to be effective in inhibiting oxidative-stress-induced apoptosis, attenuating the elevated levels of Glu and Asp induced by cerebral ischemia and resisting lipopolysaccharide (LPS)-induced inflammation [10]. Drug B, Jasminoidin, derived from the dried fruit of *Gardenia Jasminoides Ellis* and is effective to attenuate the neuronal death in the modeled ischemic environment [24]. Drug C, Desoxycholic acid, widely used in TCM, is effective to improve digestive function [28]. To illuminate the synergism and rules from combination of Baicalin, Jasminoidin and Desoxycholic acid, we employ mRNA microarray to observe the expression profiles after different therapeutic administrations. Based on our present study, we chose a custom mRNA microarray, on which 373 ischemia-related genes were selected according to the previous studies or related pathways. In this microarray, probe genes were mainly involved in cell communication, developmental process, negative regulation of biological process, cell death, wnt signaling pathway, positive regulation of biological process, catalytic activity and regulation of a molecular function. After ischemia-reperfusion, most of them were down-regulated, which is coincident with the previous studies [29]–[31]. All of our designed protocols made a majority of genes up-regulated, compared to the model group and their changing tendencies had high positive correlations with sham/model. Based on IPA software, which drawn on published, peer-reviewed literature, the bio-functions demonstrated that all kinds of designed drug-administrations, especially ABC combinations, had positive effects in regulating gene expressions after ischemia-reperfusion. However, this analysis might not tell us the essence of the molecular mechanisms of combinative effects, considering that we could not find any relations in these quantities and pharmacodynamic results. It was ambiguous to make clear the quantitative correlation between genes performance and therapeutic effects.

As has been suggested in western medicine studies, different composition materials may elicit different biochemical responses and they are thus intentionally used to regulate the corresponding physiological mechanisms. Consequently, the measured effects in genes usually combine several independent unique drug effects. Since the aim of this study is to investigate the combinational effects of drugs, it is reasonable to decompose the representation features of multiple genes into several functional groups that are sensitive to different combination of drugs. In bioinformatics, Principal component analysis (PCA) is an efficient and effective feature selection method, which has been widely used in a variety of scientific fields to extract the common patterns from multiple observation samples. Hence, PCA is a perfect tool for our purpose, whose power has been validated by previous genomic studies [32], [33]. In the present study, the top five principal components (PCs) could explain more than 95% of variation of gene profiles in different experimental groups and were selected to analyze.

**Figure 6:**
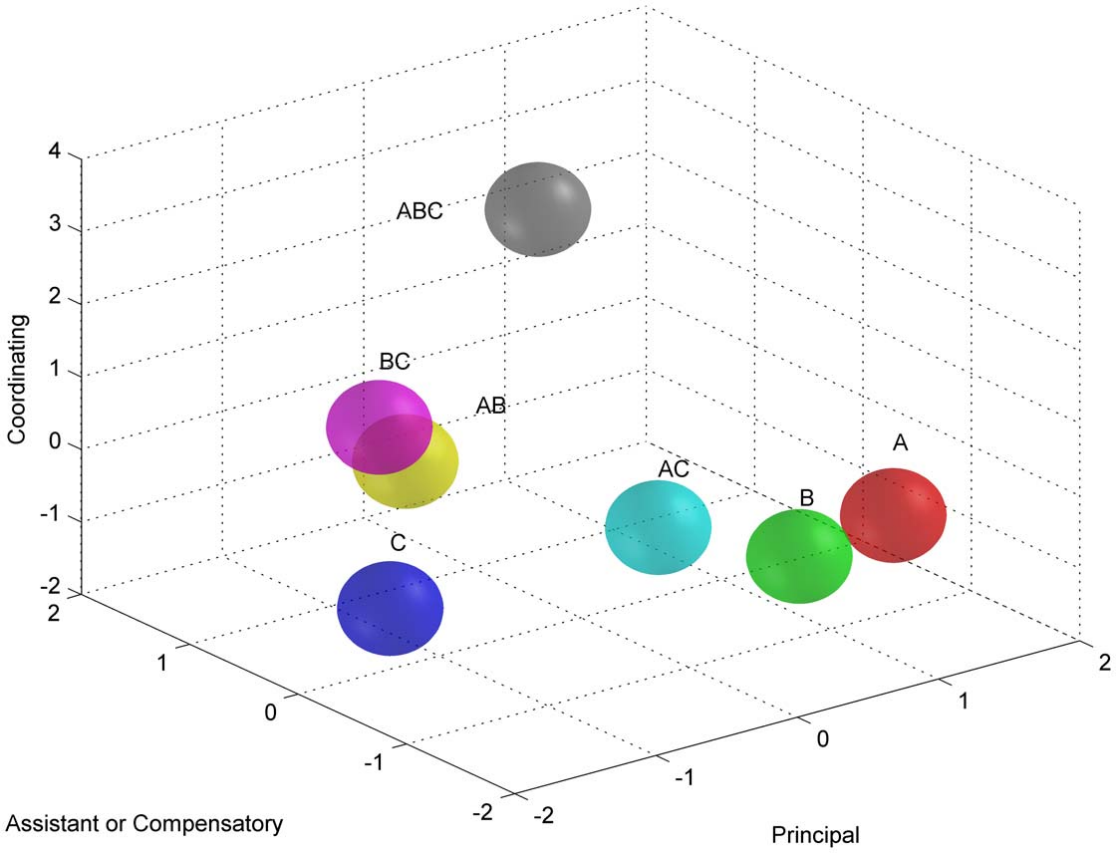
Distribution of experimental groups in the theoretic model of TCM combined therapy. The three axes in the 3D space reflect three emphases of TCM combined therapy, principal therapeutic potency, assistant or compensatory helper and compatibility of a formula. In the present study, group A displayed a greatest principal therapeutic potency, but group ABC achieved a maximized equilibrium for the three aspects.

In principal gene analysis of PCA, the attention was shifted to the PCA values of the five Principal Components (PCs) in separate experimental groups. When considering the PC1, it was clear to find a general inclination that groups administrated by Baicalin (A, AB, and ABC) showed higher values than others, while groups administrated by Desoxycholic acid showed lowest values (Fig. 5). In our anticipation, Baicalin, play the leading role in the formula, represents a most powerful therapeutic potency in the treatments, while Desoxycholic acid, playing a compatible role, as supposed nearly has no therapeutic effect. This anticipation is strongly supported by a mass of evidences [34]–[36]. For the dominant genes and involved cell processes, PC2 and PC5 had similarities in quantities and distributions. The rest PC3 and PC4 had obvious commonness. That is, they both showed highest PCA values in ABC, the best curative protocol, and they had the similar quantities of dominant genes and share most of involved cell processes. However, the PCA values of the PCs in experimental groups were ambiguous to comprehend until they were added together. The accumulation of values of the two provided a clear inclination. That is, when Baicalin existed, combinations were better than single administrations, and the performances in combined groups were influenced by the grades of the separate herbal components and their dosages. Therefore, we also considered the additive form of their values and found that except the polarized ABC and AB, rest experimental groups nearly had a same performance (Fig. 6). This manifestation just accorded with the principle of herbal formulae (Fang-Ji), emperor-minister-adjuvant-courier (Jun-Chen-Zuo-Shi) [37]. In a traditional formula, emperor aims at the cardinal pathological symptom of a disease, playing a key role in the treatments. Minister may assist emperor or treat other secondary symptoms, if applicable. Adjuvant/courier mainly co-ordinates the formula, facilitating performances of emperor and minister and decreasing their side effects. According these, in refined QKL, Baicalin is the emperor; Jasminoidin might be as the minister, and Desoxycholic acid used in the compounds as the adjuvant/courier.

In the term of genes' direct expressions, from dominant genes of PC1, we screened seven genes which manifested the highest ratios in group A among all the experimental groups, to analyze the specific potency of Baicalin, the emperor of the formula. Interestingly, the seven genes, BDNF, CRKL, FZD7, RARA, RARB, RGS5 and TGF-β1, are all specific in PC1 but have a wide-range distribution in cell. BDNF and TGF-β1, the growth factors located in extracellular matrix, both play a comprehensive role in neuroprotection after ischemia through ERK pathway or SMAD family, respectively as the important extracellular signals [38], [39]. RARA, RARB and FZD7, the receptors located in plasma member, participated in receiving signaling of anti-oxidant injury and vascular development [40], [41]. Located in cytoplasm, CRKL and RGS5 play a role in nerve system development [42], [43]. Noticeably, BDNF and TGF-β1, the two essential factors in the neuroprotective function, showed a decreasing inclination as the doses of Baicalin decreased in experimental groups, which implied the Baicalin's potency as emperor in the formula. In the dominant genes of PC 3 and PC 4, we screened eleven genes showed the highest ratios in the group ABC among all the experimental groups, to study the integrated effect of ABC. Among these genes, ten shared with other types of PCs, while only ELK3, a transcription factor which has been reported to be important in vascular development [44], was specific in PC3 and PC4. Furthermore, SHC1 forms an important receptor protein, participating in some anti-ischemic processes, such as proliferation and cell survival. However, it was also reported to catalyze formation of ROS, related to reperfusion damage [45]. SMAD3, BARHL1 and CREM are all transcription factors, playing roles in cell differentiation and proliferation. Especially, SMAD3 and members of its family, transducting the signaling of TGF-β, are the important protective factor in ischemia/reperfusion [38]. ADCY3 is an adenylate cyclase, catalyzing the formation of cAMP. NKD 1 is a dishevelled-binding protein that functions as a negative regulator of the Wnt-beta-catenin-Tcf signaling pathway [46]. HSPA1A is involved in cell attachment as a molecular chaperon, playing a role in ATP-depletion cytoprotection [47]. MGAT1 plays a major role in energy absorption, due to its function in catalyzing the synthesis of diacylglycerol, the precursor of physiologically important lipids such as triacylglycerol and phospholipids [48]. FREQ regulates exocytosis through mediating increased pohosphinositide turnover and Ca2+ signaling [49] and HTR2C is a part of serotonin receptor, both of which participate in neurotransmission. Summarily, the selected genes are evidently implicated in varied ischemia or nerve system related pathways, which suggested that the great integrated effect of ABC might be derived from the equilibrium of the multiple physiological processes, rather than a powerful therapeutic efficacy in one main process. In the PC2 and PC5, there were twenty one dominant genes showing highest ratios in group AB, so we selected them as delegates of the function of the Minister of the formula. Among extracellular factors, interleukin 1 alpha (IL1a) and colony stimulating factor 1 (CSF1) are important cytokines related to ischemic injury. IL1a was reported to enhance neuronal damage through inflammation [50], while it was also regarded to play a role in neuroprotection to excitotoxin mediated by NMDA [51]. CSF1 has been determined to be effective in neuronal survival in cerebral ischemic lesion [52]. The other two, WIF1 and DKK2, were members of Wnt pathway, which plays a role in resisting development of neuronal death [53]. There were seven proteins located in nucleus, ex. DAXX, TCF12, CASP2, TBP, HDAC1, IKBKG and Taf7. All of them participate in transcription, except CASP2, which may be involved in apoptosis, as well as BAD and CASP7 in cytoplasm. The other proteins in cytoplasm or membrane, such as TRAF2, GAK, MKNK1, Rgs6, GAB1, HTR3A and HTR1A, were mainly implicated in cellular signaling transduction. Importantly, the essential neuroprotective protein, nerve growth factor [54], also showed a dramatic increase in the AB. Summarily, the dominant genes in PC 2 and PC 5 provided extra main neuroprotective pathways other than PC 1, which suggested a compensatory role of jasminoidin as the Minister in this formula.

### Conclusion

We firstly presented the synergism and combinational rules of refined Qing-Kai-Ling to treat ischemic stroke mice model through conventional neurobiological methods combined with PCA of microarray. TCM is a comprehensive and abstruse subject, which has accumulated a mass of clinical experience but absence of a systematical theory and scientific explanation. Our approach successfully simulated the therapeutic ways by extracting principal components, according to the classic herbal drug-combination theory, emperor-minister-adjuvant-courier, which is a fundamental philosophy of TCM. This study might be considered to provide a new sight to investigate and understand the molecular mechanism of TCM, because it was a pilot attempt to explain combination theory of TCM in aspect of holism. On the other hand, we also indicated the PCA of information technology may be a potential approach to comprehend the gene profiles of combined therapy.

## Materials and Methods

### Animals handing procedure

Male KunMing mice weighing (45±5 g) were purchased from the laboratory animal center of Peking University (certificate No. 2005-0004). They were housed under standard laboratory conditions. Food and water were provided ad libitum. The middle cerebral artery occlusion reperfusion model (MCAO) was prepared according to the method described previously [25], [26]. In short, animals were anesthetized with 2% sodium pentobarbiturate solution (4 mg/kg weight, i.p.). A midline neck incision was made. The left common carotid artery, the left external carotid artery (ECA) and the left inner carotid artery were exposed. After a ligation in the distal part, an incision was made in the left external carotid near the carotid bifurcation. A 4 to 0 monofilament nylon suture coated with silicon was inserted through this incision and gently advanced into the internal carotid artery. The filament was sent into the intracranial portion of the internal carotid and positioned 11±0.5 mm from the carotid bifurcation. By this method, a large infarct in the territory of the middle cerebral artery is typically produced. Body temperature measured in the temporal muscle was maintained at 37±0.5°C with a heated water-blanket under feedback control during the period of ischemia. Reperfusion was performed by withdrawal of the inserted filament after 1.5 hours. Animals in the sham-operation group underwent the same procedures as described above with the exception of the insertion of the nylon filament into the inner carotid. 10 mice were randomly selected and assigned to the sham-operation group (N). 176 mice survived from the MCAO operation were randomly assigned to rest 8 groups: (1) the MCAO model group (M), (2) the baicalin-treated group (A), (3) the jasminoidin-treated group (B), (4) the desoxycholic acid-treated group (C), (5) the combination of baicalin and jasminoidin-treated group (AB), (6) the combination of baicalin and desoxycholic acid-treated group (AC), (7) the combination of jasminoidin and desoxycholic acid-treated group (BC) and (8) the combination of baicalin, jasminoidin and desoxycholic acid-treated group (ABC). All the animal experiments were performed according to the Prevention of Cruelty to Animals Act 1986 and NIH Guidelines for Care and Use of Laboratory Animals and local laws. People who analyzed the subsequent data did not know this assignment.

### Drug administration

Baicalin, jasminoidin and desoxycholic acid were kindly supplied by the pharmaceutical factory affiliated with Beijing University of Chinese Medicine. The individual ingredients in refined QKL are baicalin 6.25 g/L, jasminoidin 12.5 g/L, and desoxycholic acid 7 g/L [9]. According to the results of initial experiments on the dosage selecting of QKL (data not show), we determined individual into animal administration dosage, i.e. baicalin (20 mg/kg) for A group, jasminoidin (100 mg/kg) for B group, and desoxycholic acid (28 mg/kg) for C group. In order to highlight the synergism of combinational drugs, the half of dosage was tested for combination of pairs, and one third of dosage was tested for combination of all, i.e. baicalin (10 mg/kg) and jasminoidin (50 mg/kg) mixture for AB group, baicalin (10 mg/kg) and desoxycholic acid (14 mg/kg) mixture for AC group, jasminoidin (50 mg/kg) and desoxycholic acid (14 mg/kg) mixture for BC, baicalin (6.67 mg/kg), jasminoidin (33.3 mg/kg) and desoxycholic acid (9.3 mg/kg) mixture for ABC group. The drugs were administrated in saline and injected from the tail vein (i.v.) with 4 ml/kg solution just before reperfusion. Animals in the sham operation group and the MCAO model group received an injection of 4 ml/kg saline with the same method as mentioned above.

### Neurological examination

24 hours after reperfusion, sixteen mice of each group were subjected to a neurological examination according to the method established by Bederson, et al [27]. The postural reflex test and the forelimb placing test were performed. The neurologic deficit degree was graded by 0 to 3 score (normal = 0; the worst = 3).

### TTC staining

24 hours after reperfusion, in each group, the sixteen mice for neurological examination were anesthetized with chloral hydrate (400 mg/kg) and decapitated. The brains were removed and sectioned into five 2 mm-thick coronal slices. The slices were stained with 1% of 2,3,5-triphenyltertrazolium chloride (TTC, Sigma) in 0.1 M PBS (pH 7.4) for 30 minutes, room temperature (RT), and fixed with 10% buffered formalin overnight. They were photographed with a digital camera (Color CCD camera TP-6001A, Topica). The infarct volumes were measured by Pathology Image Analysis System (Topica Inc, Japan). And in each sample, the total infarction volume and the total slice volume were collected from the five slices, and then the infarction volume ratio was calculated as dividing the total infarction area by the total slice area.

### Microarray Experiments and data analysis

A cDNA microarray was manufactured by mechanical spotting on glass slides with Array Spotter Generation III (Molecular Dynamics Inc.). This microarray contained 373 cDNA derived from a cDNA library (Invitrogen, Cat.1065-025). Each clone was sequence-verified, whereas each cDNA represented one gene. These genes were selected as ischemia-related genes concluded from our previous work [55] and other published papers, which included the genes related to the signal transduction pathway [56]–[60] and cascade reaction corresponding to cerebral ischemia combined with neural protection and injured factors [61]–[63]. Three animals in each group were randomly selected to extract RNA. Three hours after reperfusion, animals were decapitated, and the brains were removed under RNAse-free conditions. The left cerebral hemisphere was carefully dissected out. Tissues were placed into Trizol reagent (Life Technologies, Gibco, Rockville, MD), and then homogenized. Total RNA was extracted with chloroform and isopropyl alcohol and purified with a RNAeasy kit (Qiagen, Valencia, CA). The quality of RNA was ensured with an eletrophoresis in 1% agarose gel.

RNA of the three samples in the MCAO group was pooled as one. For each microarray analysis, 50 µg total RNA of each sample was used. cDNA was reverse-transcripted from RNA and labelled with Cy5(MCAO group) or Cy3(drug treated or sham operation groups), with ampliscribe T7(Epicenter, Madison, WI, USA). cDNA was then hybridized to the microarray at 42°C in a buffer containing 50% formamide, 5×SSC, 0.1%SDS, 0.25 µg/µl human cotl DNA and 0.125 µg/µl poly-dA, for 16 hours. The hybridized chip was washed with a series of SSC/SDS solutions. After washing, slides were immediately scanned on an Axon Gene Pix 4000B and quantified using Axon GenePix Pro (Axon, Union city, CA, USA). Data were analyzed using GeneSpring (Silicon Genetics, Redwood City, CA, USA). The data were normalized through housekeeping gene correction and LOWESS method. At last, there were three independent microarray results in each drug-treated group. We compared gene expressions in drug-treated groups with that in the MCAO group, as well as compared gene expressions among drug-treated groups.

### Real-time RT-PCR Analysis

Real-Time PCR (50 µl) was performed with Thermo-fast 96 PCR plates(Bio-Rad Laboratories, Hercules, Calif.), which were sealed with iCycler IQ optical-quality tapes (Bio-Rad Laboratories) on an iCycler IQ thermocycler (Bio-Rad Laboratories). Each measurement was performed in three replicates. A dilution series of positive control DNA was used inthe same plates as the calibration standards. Positive control DNA was generated by the amplification of the gene from samples. The amplicon was purified, cloned into TOPO TA cloning vector (Invitrogen), and reamplified. After purification, the concentrations of PCR products were determined using a fluorometer as described above and were serially diluted to generate calibration standards. Data analysis was carried out with iCycler software (Bio-Rad Laboratories). Based on the standard curve, a threshold cycle measured in a sample was converted to the copy number of the gene in a sample.

### Statistical and gene expression profile Analysis

Data were represented as mean±SD. Data were analyzed with SPSS 17.0 software. In all the cases, One-Way ANOVA and t test were used for comparisons between multiple groups and two groups, respectively. P<0.05 was considered as statistically significant.

Gene data were analyzed through the use of Ingenuity pathway Analysis (IPA), Ingenuity systems, www.ingenuity.com. In brief, a data set containing gene identifiers and corresponding expression fold change values was uploaded into the application. Each gene identifier was mapped to its corresponding gene object in the ingenuity pathway knowledge base.

The principal component analysis was run following the algorithms. Briefly, Let 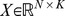 be the gene data from xxx subjects with N genes and K experimental conditions. In our study, we mainly recorded 373 representations in gene chips in 7 different combinations of three drug components (A, B and C). Before feature selection, the gene values from multiple subjects are firstly averaged to enhance the data quality. We used 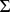 to represent the covariance of 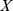 and PCA essentially pursuits the following eigenvalue decomposition problem
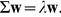
(1)


Here, 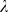 is the eigenvalue of X. We selected the largest five eigenvalues whose overall contribution is 96.89%. They represent the five most important drug functions. 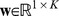 is a calculated eigenvector which can depict the contribution of every gene to one functional component. After normalizing each vector, we combined them and generated a projection matrix 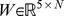. Then 373 genes values were transformed to five principal response components in 7 conditions by applying W to the gene data
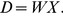
(2)


In the aspect of principal gene analysis, we accumulated and normalized one gene's PCA scores of all the experimental groups, to investigate the power of a gene in a PC. Then we set a threshold of ±0.2 to screen the dominant genes in a PC, because in any PC, most of genes were normally distributed between −0.2–0.2 with a minority of influence but genes distributed outside of −0.2–0.2 had a majority of influence to the PC. To annotate function of genes, we applied Pathway studio 5.0 (updated pathway in Dec 7th, 2010) to analysis the outcome of each PC.

## Supporting Information

Figure S1 **Validation of microarray results (tiff).** The real-time PCR results figured out the similar profiles with the outcome of microarray. The results suggested that array based gene expression measurements were reliable. Noted: blue columns show the results of real time PCR; red columns show microarray results.
(TIF)

Table S1 **Selected 46 drug-target genes (excel).** The yellow highlight show the common genes affected by each combination of drugs.
(DOC)

Table S2 **The dominant genes in the five principal components (PCs) (excel).** A gene whose absolute normalized score in a PC more than 0.2 was selected as a dominant gene. A colorful dot means that the gene is a dominant gene in the corresponding PC.
(DOCX)
