# Supplementary figures and images for: Synergism and Rules from Combination of Baicalin, Jasminoidin and Desoxycholic acid in Refined Qing Kai Ling for Treat Ischemic Stroke Mice Model
Source: PLoS One. 2012 Sep 26;7(9):e45811. doi: 10.1371/journal.pone.0045811 (PMC3458908; doi:10.1371/journal.pone.0045811)

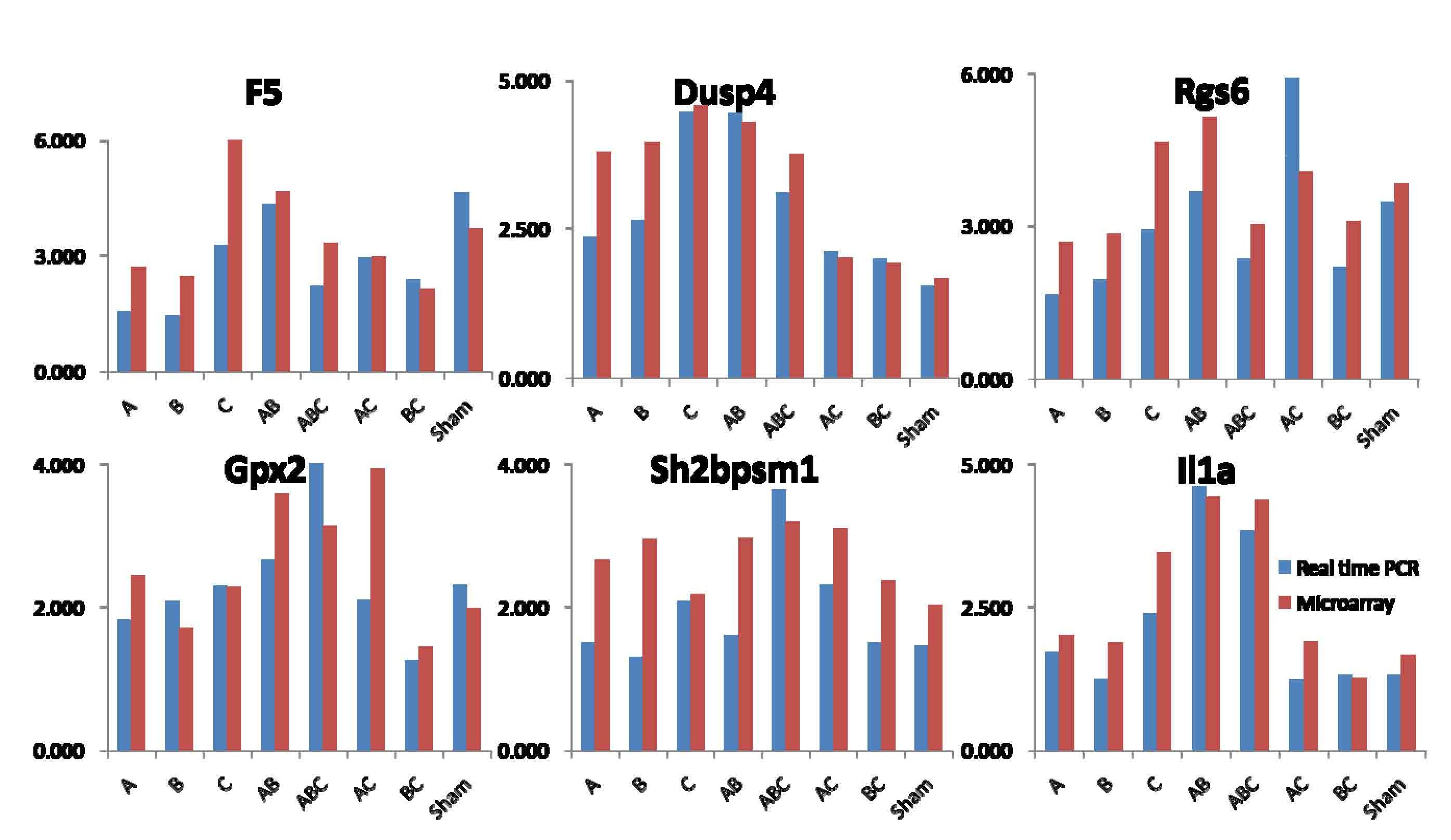

Supplement: Figure S1 — Validation of microarray results (tiff). The real-time PCR results figured out the similar profiles with the outcome of microarray. The results suggested that array based gene expression measurements were reliable. Noted: blue columns show the results of real time PCR; red columns show microarray results. (TIF) [file pone.0045811.s001.tif]
