# Supplementary material for: Synergism and Rules from Combination of Baicalin, Jasminoidin and Desoxycholic acid in Refined Qing Kai Ling for Treat Ischemic Stroke Mice Model
Source: PLoS One. 2012 Sep 26;7(9):e45811. doi: 10.1371/journal.pone.0045811 (PMC3458908; doi:10.1371/journal.pone.0045811)
Supplement: Table S1 — Selected 46 drug-target genes (excel). The yellow highlight show the common genes affected by each combination of drugs. (DOC) [file pone.0045811.s002.doc]

Table S1. Selected 46 drug-target genes

| drug A | drug B | drug C | drugAB | drugAC | drugBC | drugABC |
| --- | --- | --- | --- | --- | --- | --- |
| ADORA1 | ADORA1 |  |  | ADORA1 | ADORA1 | ADORA1 |
| BRAF | BRAF | BRAF |  | BRAF |  | BRAF |
| CASP3 | CASP3 |  | CASP3 | CASP3 |  | CASP3 |
|  |  | CDK4 | CDK4 | CDK4 |  | CDK4 |
|  |  | EGFR | EGFR | EGFR |  | EGFR |
| GRIN2B |  |  |  | GRIN2B |  | GRIN2B |
| GRIN1 | GRIN1 | GRIN1 |  | GRIN1 | GRIN1 | GRIN1 |
| HTR1A | HTR1A | HTR1A | HTR1A | HTR1A | HTR1A | HTR1A |
| HTR1F | HTR1F | HTR1F | HTR1F | HTR1F | HTR1F | HTR1F |
| HTR2C | HTR2C | HTR2C | HTR2C | HTR2C | HTR2C | HTR2C |
| HTR3A | HTR3A | HTR3A | HTR3A | HTR3A | HTR3A | HTR3A |
| HTR7 | HTR7 | HTR7 | HTR7 | HTR7 | HTR7 | HTR7 |
| IL1A | IL1A | IL1A | IL1A | IL1A | IL1A | IL1A |
| VEGFA | VEGFA | VEGFA | VEGFA | VEGFA | VEGFA | VEGFA |
| TOP2B | TOP2B | TOP2B | TOP2B | TOP2B | TOP2B | TOP2B |
| SRC | SRC | SRC | SRC | SRC | SRC | SRC |
| RARB | RARB | RARB | RARB | RARB | RARB | RARB |
| CCR5 | CCR5 | CCR5 | CCR5 | CCR5 | CCR5 | CCR5 |
| CDK5 | CDK5 | CDK5 | CDK5 | CDK5 | CDK5 | CDK5 |
| F5 | F5 | F5 | F5 | F5 | F5 | F5 |
| CACNA1B | CACNA1B | CACNA1B | CACNA1B | CACNA1B | CACNA1B | CACNA1B |
| IFNAR2 |  | IFNAR2 | IFNAR2 | IFNAR2 |  |  |
|  |  | IFNAR1 | IFNAR1 | IFNAR1 |  | IFNAR1 |
| IL6R |  | IL6R | IL6R | IL6R |  | IL6R |
| IL7R | IL7R | IL7R | IL7R | IL7R |  | IL7R |
| KCNMB1 | KCNMB1 | KCNMB1 | KCNMB1 | KCNMB1 |  | KCNMB1 |
| PARP1 | PARP1 | PARP1 | PARP1 | PARP1 |  | PARP1 |
| RELA |  | RELA | RELA | RELA |  | RELA |
| RARG |  | RARG |  | RARG |  | RARG |
|  | PIK3CA | PIK3CA | PIK3CA |  |  | PIK3CA |
| GRIN2A |  |  |  | GRIN2A |  | GRIN2A |
| TGFB2 | TGFB2 |  | TGFB2 | TGFB2 | TGFB2 | TGFB2 |
| TUBA1A | TUBA1A |  |  |  |  |  |
|  | PRKCB |  |  |  |  |  |
|  |  | PRKCA | PRKCA |  |  |  |
|  |  | TUBB3 |  |  |  |  |
|  |  |  | MAP2K2 | MAP2K2 |  | PRKCA |
|  |  |  |  | CSF2RB |  |  |
|  |  |  |  |  |  | IL1B |
|  |  |  |  |  | RAF1 | RAF1 |
|  |  |  |  |  | MAP2K1 |  |
|  |  |  |  |  |  |  |
