# Supplementary material for: Synergism and Rules from Combination of Baicalin, Jasminoidin and Desoxycholic acid in Refined Qing Kai Ling for Treat Ischemic Stroke Mice Model
Source: PLoS One. 2012 Sep 26;7(9):e45811. doi: 10.1371/journal.pone.0045811 (PMC3458908; doi:10.1371/journal.pone.0045811)
Supplement: Table S2 — The dominant genes in the five principal components (PCs) (excel). A gene whose absolute normalized score in a PC more than 0.2 was selected as a dominant gene. A colorful dot means that the gene is a dominant gene in the corresponding PC. (DOCX) [file pone.0045811.s003.docx]

**Table S2. The dominant genes in the five principal components.**

| **Dominant Gene** | PC1 | PC2 | PC3 | PC4 | PC5 |
| --- | --- | --- | --- | --- | --- |
| **Apoe** | **●** | **●** | **●** | **●** | **●** |
| **Gab1** | **●** | **●** | **●** | **●** | **●** |
| Adcy3 | ● |  | ● | ● | ● |
| Barhl1 |  | ● | ● | ● | ● |
| Casp7 | ● | ● | ● |  | ● |
| Dusp4 | ● | ● | ● |  | ● |
| F5 | ● | ● | ● |  | ● |
| Gak | ● | ● | ● |  | ● |
| Htr3a | ● | ● | ● |  | ● |
| Ikbkg |  | ● | ● | ● | ● |
| Mogat1 | ● |  | ● | ● | ● |
| Rgs6 | ● | ● | ● |  | ● |
| Tbp |  | ● | ● | ● | ● |
| Adcyap1r1 | ● |  | ● | ● |  |
| Adora1 | ● |  | ● | ● |  |
| Casp2 |  | ● |  | ● | ● |
| Daxx | ● |  |  | ● | ● |
| E2f1 | ● |  | ● | ● |  |
| Fadd |  | ● |  | ● | ● |
| Freq | ● | ● |  | ● |  |
| Grin1 |  | ● | ● | ● |  |
| Htr1a | ● | ● |  |  | ● |
| Htr1f | ● |  |  | ● | ● |
| Htr2c |  | ● | ● |  | ● |
| Pin1 | ● | ● |  | ● |  |
| Plcg2 | ● | ● | ● |  |  |
| Shc1 | ● | ● |  | ● |  |
| Taf7 | ● |  |  | ● | ● |
| Top2b | ● |  | ● | ● |  |
| Bad | ● | ● |  |  |  |
| Crem | ● |  | ● |  |  |
| Csf1 |  | ● |  |  | ● |
| Dgka | ● | ● |  |  |  |
| Fzd10 | ● |  |  | ● |  |
| Gna14 | ● | ● |  |  |  |
| Hdac1 | ● |  |  |  | ● |
| Hspa1a | ● |  |  | ● |  |
| Matn2 | ● |  |  |  | ● |
| Mlh3 | ● |  | ● |  |  |
| Nkd1 |  |  | ● |  | ● |
| Pold2 | ● | ● |  |  |  |
| Smad3 | ● |  |  | ● |  |
| Tcf12 |  | ● | ● |  |  |
| Traf2 |  |  |  | ● | ● |
| Adamts1 | ● |  |  |  |  |
| Ak1 | ● |  |  |  |  |
| Bdnf | ● |  |  |  |  |
| Bmp1 |  | ● |  |  |  |
| Crkl | ● |  |  |  |  |
| Dkk2 |  |  |  |  | ● |
| Eef2k | ● |  |  |  |  |
| Elk3 |  |  |  | ● |  |
| Fosb | ● |  |  |  |  |
| Fzd7 | ● |  |  |  |  |
| Gpx2 |  | ● |  |  |  |
| Il1a |  |  |  |  | ● |
| Kcnq1 |  | ● |  |  |  |
| Met | ● |  |  |  |  |
| Mknk1 |  |  |  |  | ● |
| Mmp2 | ● |  |  |  |  |
| Ngfg |  |  |  |  | ● |
| Pdcd11 |  | ● |  |  |  |
| Pxn | ● |  |  |  |  |
| Rara | ● |  |  |  |  |
| Rarb | ● |  |  |  |  |
| Rgs19 | ● |  |  |  |  |
| Rgs5 | ● |  |  |  |  |
| Src | ● |  |  |  |  |
| Stat5a |  | ● |  |  |  |
| Tgfb1 | ● |  |  |  |  |
| Tradd | ● |  |  |  |  |
| Wif1 |  |  |  |  | ● |
|  | | | | | |
